# Optimization of the Fine to Coarse Aggregate Ratio for the Workability and Mechanical Properties of High Strength Steel Fiber Reinforced Concretes

**DOI:** 10.3390/ma13225202

**Published:** 2020-11-17

**Authors:** Mohammad Iqbal Khan, Wasim Abbass, Mohammad Alrubaidi, Fahad K. Alqahtani

**Affiliations:** Department of Civil Engineering, College of Engineering, King Saud University, P.O. Box 800, Riyadh 11421, Saudi Arabia; wabbass@ksu.edu.sa (W.A.); en_moh88@hotmail.com (M.A.); bfahad@ksu.edu.sa (F.K.A.)

**Keywords:** steel fiber reinforced concrete, mix proportioning, workability, FA/CA ratio

## Abstract

High-strength concrete is used to provide quality control for concrete structures, yet it has the drawback of brittleness. The inclusion of fibers improves the ductility of concrete but negatively affects the fresh properties of fiber-reinforced concrete. The effects of different fine to coarse aggregate ratios on the fresh and hardened properties of steel fiber reinforced concrete were investigated in this study. Mixtures were prepared with various fine to coarse aggregate (FA/CA) ratios incorporating 1% steel fiber content (by volume) at constant water to cement ratio. The workability, unit weight, and temperature of the concrete in the fresh state, and the mechanical properties of steel-fiber-reinforced concrete (SFRC) were investigated. The inclusion of fiber in concrete influenced the mobility of concrete in the fresh state by acting as a barrier to the movement of coarse aggregate. It was observed that the concrete with an FA/CA ratio above 0.8 showed better flowability in the fresh state, whilst an above 0.9 FA/CA ratio requires excessive superplasticizer to maintain the flowability of the mixtures. The compressive and flexural strength of SFRC increased with an increase in the FA/CA ratio by around 10% and 28%, respectively. Experimental values of compressive strength and flexural strength showed good agreement, however, modulus of elasticity demonstrated slightly higher values. The experimentally obtained measurements of the mechanical properties of SFRC conformed reasonably well with the available existing prediction equations, and further enabled establishing predictive isoresponse interactive equations within the scope of the investigation domain.

## 1. Introduction

The utilization of high-strength concrete (HSC) has become an integral part of modern day construction, due to its excellent behavior in both mechanical and durability related properties. However, HSC is brittle in nature compared to normal-strength concrete (NSC). Silica fume (SF) is one of the essential materials included in HSC mixtures, however, there was no significant difference between densified SF and undensified SF in the properties of fresh and hardened ultra-high performance fiber-reinforced concrete [1]. However, inclusion of fibers reduces the workability and improves the hardened properties of the concrete. HSC with steel fibers significantly enhance the ductility, because steel fibers tend to improve the bond with the dense and well-compacted cement matrix of HSC. Usually, a high degree of vibration is required for normal fiber reinforced concrete. Better dispersion of fibers can be achieved by using self-compactible concrete, which might enhance the performance of a composite. The flow behavior of self-compacting concrete (SCC) with fibers is different compared to that of SCC without fibers [2,3,4,5]. Many researchers have found that the workability of SCC also decreases, and the flow time increases, with the addition of fibers [6,7]. Moreover, plastic viscosity increases with an increase in fiber content [8]. There are many other factors which influence the overall mechanical, durability, and microstructure properties of concrete containing fibers [9]. Design modifications in formulating a tailored high performance have been also suggested [10]. However, better dispersion of fibers is a good start to achieve beneficial advantages in both the fresh and hardened state. Composite materials containing water, cement, and aggregates with randomly dispersed fibers are known as steel fiber reinforced concrete (SFRC) [11].

Steel fibers of various shapes and sizes can be produced as specified by ACI 544.1R [12]. The concrete ductility is enhanced by crack occurrence delay or crack growth reduction. Thus, the addition of steel fibers improves the tensile strength and toughness of concrete [13,14]. The properties of fresh and hardened of SFRC are greatly influenced by its water content, cement content, and the features of the aggregates [15]. The effects of four FA/CA ratios and two different *D_max_* values on the mechanical properties of SFRC were investigated by Chenkui and Guofan [16]. It was concluded that the inclusion of steel fibers and a high value of *D_max_* led to improvements in all strength values. In another study, various fiber types with different groups of aggregate were used to make SFRC mixtures. The bond strength was improved in these mixtures by increasing the fine aggregate (FA)-to-coarse aggregate (CA) ratio [17]. Thus, the proportion of FA and CA included affects the fresh and hardened properties of the SFRC. The ACI committee 544 recommends the use of aggregate gradations for trial mixtures and *D_max_* values [11]. For the design of SFRC, the fiber content and type, aggregate content, and proportion of fine aggregate are key parameters. ACI 318 code [18] recommends incorporating 1% of volume fraction of hooked-end fibers to improve the ductile flexural behavior. The incorporation 0.98‒1.10% of hooked-end fibers was required to satisfy the minimum reinforcement ratio [19], and 1.0% volume fraction of fiber is required to achieve a multiple cracking behavior [20].

Coarse and fine aggregate contents affect a given dosage of fiber content in SFRC more significantly. Several researchers have introduced different mixture design approaches based on optimization of the packing density, and some of these researchers studied the effects of the contents of cement and water on the mechanical properties of SFRC [11,14,15,21,22]. However, there is limited literature available on the use of fine to coarse aggregate ratios for a mixed design for FRC. The maximal fine to coarse aggregate ratio described in ACI 544.3R-2008 [23] is 0.6. Coarse and fine aggregate comprises almost up to 75% of total concrete volume, therefore, balancing the usage of FA and CA plays vital role in determining the performance and quality of the concrete. Therefore, more studies are required to determine the effects of an increased FA/CA ratio on fiber reinforced concrete. This research work was conducted to arrive at an optimum FA/CA, in terms of the properties of both fresh and hardened concrete. The results obtained demonstrated that FA/CA ratios can affect the freshness and mechanical properties of concretes with similar paste volumes.

## 2. Experimental Program

### 2.1. Materials

Ordinary Portland cement (OPC), conforming to ASTM type-I, was used for the preparation of all mixtures, and satisfies the ASTM C150 specifications [24]. The chemical composition and the physical properties of OPC are presented in Table 1.

Glenium 51 produced by BASF (Ludwigshafen, Germany), a polycarboxylic ether-based structure superplasticizer (SP), satisfying the ASTM C494 type F specifications [25], was used in this investigation. The superplasticizer dosage was used as a dry extract as a percentage of the cement weight.

**Figure 1:**
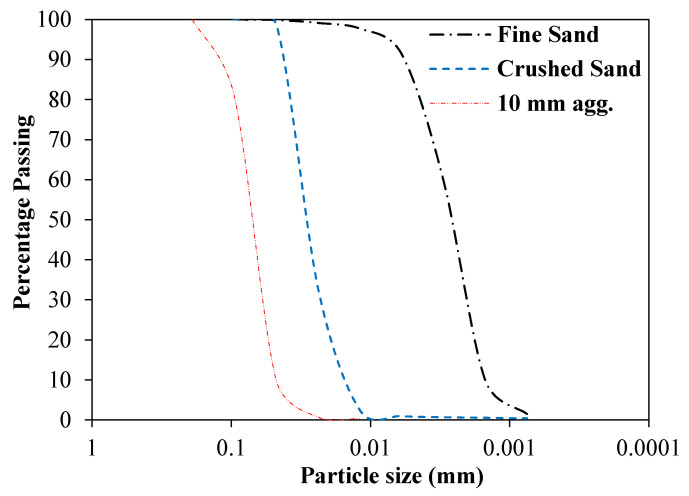
Distribution of particle size for fine and coarse aggregate (FA/CA).

FA with a size of 0–3 mm and CA with a nominal grain size of 10 mm, conforming to ASTM C33, [26] was used. The particle size distribution of FA and CA is shown in Figure 1. The FA and CA had specific gravities and water absorption capacities: 2.70% and 1.45% and 2.65% and 0.75%, respectively. A fineness modulus of 2.54 was attained by a suitable combination of fine and crushed sand.

**Figure 2:**
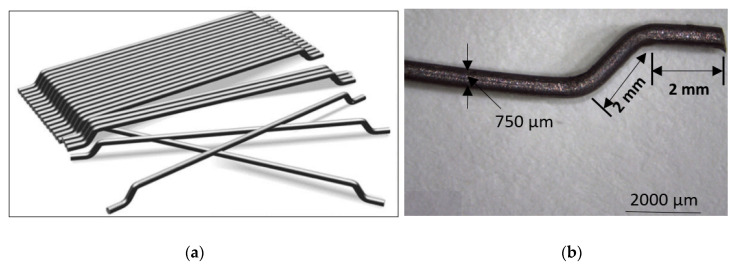
Steel fibers used in this investigation (**a**) typical form (**b**) optical microscope picture.

Hooked-ended steel fibers used in this investigation were as given in Figure 2, while their physico-mechanical properties are presented in Table 2. Hooked-ended steel fibers of 1% volume fraction were used, based on the recommendations of the available literature [18,19,20].

### 2.2. Mix Proportions and Sample Preparation

A total of sixteen SFRC mixtures with different FA/CA ratios were prepared. Eight mixtures were without fibers, and eight with fibers. The volume fractions of steel fibers, cement, and water, as well as the total aggregate content, were kept constant. In the mix design, a cement content of 500 kg/m^3^, a water-to-cement (w/c) ratio of 0.30, and a fiber content of 78.5 kg/m^3^ were used. The SP dosage was optimized for each mixture to assess its targeted workability. A series of mixtures were prepared with different FA/CA ratios of 0.50, 0.60, 0.70, 0.80, 0.90, 1.0, 1.10, and 1.20 to determine the optimal dosage of SP. The mixture proportions are presented in Table 3.

All aggregates were mixed in a dry state with the addition of water for absorption, followed by the addition of cement. The constituents were mixed for one minute and 70–80% of the water was added and mixed for 1 min. After that, the rest of the water along with the SP was added to the mixture, and mixing continued for 3 min, followed by a rest of 3 min. Then, lastly, mixing occurred for 2 min. Fibers were added at the end and mixed until the fibers were well dispersed in the mixture. Then, the slump was measured to evaluate the horizontal flow of FRC. The mixtures were designated as M50, M60, etc., where 50 represents an FA/CA ratio of 0.5.

Casting and compacting was conducted in accordance with ASTM C31 [27]. After the casting, the specimens were covered under damp hessian and polyethylene sheets for 24 h. The demolding of the specimens was carried out on the following day, and then they were placed in a mist room at 20 ± 2 °C and 98 ± 2% RH until testing.

The testing of all mixtures, with and without fibers, was performed in both fresh and hardened states. The workability, unit weight, and temperature were measured in the fresh state, whereas the compressive strength, the modulus of elasticity, and the flexural strength were investigated in the hardened state.

### 2.3. Testing Procedure

An experimental investigation with two steps was designed. In the first step, the slump of concrete (with and without fiber), the unit weight, and the temperature were measured to investigate the influence of the addition of steel fibers in the fresh state. At the second step, the mechanical properties of the concrete were investigated. All samples were stored in a tank of lime saturated water at 20 ± 2 °C until testing. To evaluate the workability of the fresh concrete, a truncated cone apparatus, with a cone diameter of 100–200 mm and height of 300 mm, was used. This cone was centered on the base plate and, after the filling of the cone, this cone was lifted vertically upward, and the drop in the height of the concrete was measured as the slump.

A 100 × 200 mm cylinder was used to measure the uniaxial compressive strength. A compressometer set up was used to measure the modulus of elasticity of the concrete. The modulus of elasticity was measured as per the ASTM C469 [28] method using the following formula:

(1) $$E_{c}=\frac{\left(S_{2}-S_{1}\right)}{\left(\varepsilon_{2}-0.000050\right)}$$

where: *Ec* is modulus of elasticity (psi), *S*_2_ is stress corresponding to 40% of ultimate load, *S*_1_ is stress corresponding to a longitudinal strain, *ε*_1_, of 50 millionths (psi), and *ε*_2_ is longitudinal strain (produced by stress *S*_2_).

**Figure 3:**
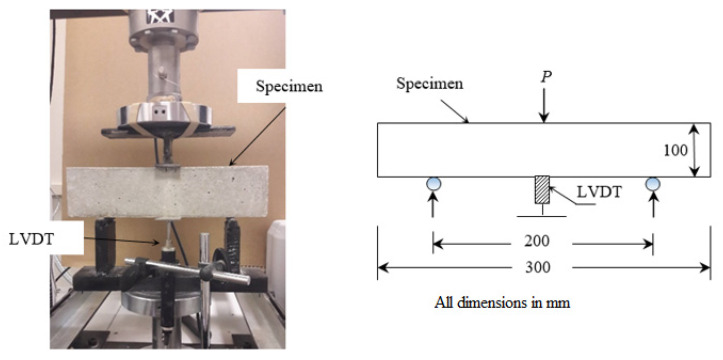
Three-point flexural test setup.

Prismatic samples 100 × 100 × 300 mm were tested at a loading rate of 0.2 mm/min, with third point loading using the set up shown in Figure 3, to measure the flexural strength of all the mixes. The results of the mechanical properties are presented as the average result from three samples.

### 2.4. Prediction Models

Based on the experimental results, quadratic interactive response surface models were developed using Minitab software [29]. These models have predictive capabilities, and these were used to for creating isoresponse contour curves from the parameters within the experimental domain of the investigation. Prediction models for the slump, compressive strength, modulus of elastic, and flexural strength of the concrete were developed.

A responsive variable f(x) is measured with a two-factor interactive effect $x_{1}$ (FA content) and $x_{2}$ (CA content) using the following model:

(2) $$f(x)=\beta_{0}+\beta_{1}x_{1}+\beta_{2}x_{2}+\beta_{11}x_{1}^{2}+\beta_{22}x_{2}^{2}+\beta_{12}x_{1}x_{2}$$

where f(x) is the responsive variable; $x_{1}$ and $x_{2}$ are the experimentally measured factor variables for f(x); $\beta_{0}$, $\beta_{1}$, $\beta_{2}$, …, $\beta_{12}$ are the coefficients of the model.

## 3. Results and Discussion

### 3.1. Properties of Fresh Concrete

The properties of the fresh concrete measured as slump (with and without fiber content), temperature, and unit weight are presented in Table 4.

#### 3.1.1. Influence of Steel Fibers on Slump of Concrete

**Figure 4:**
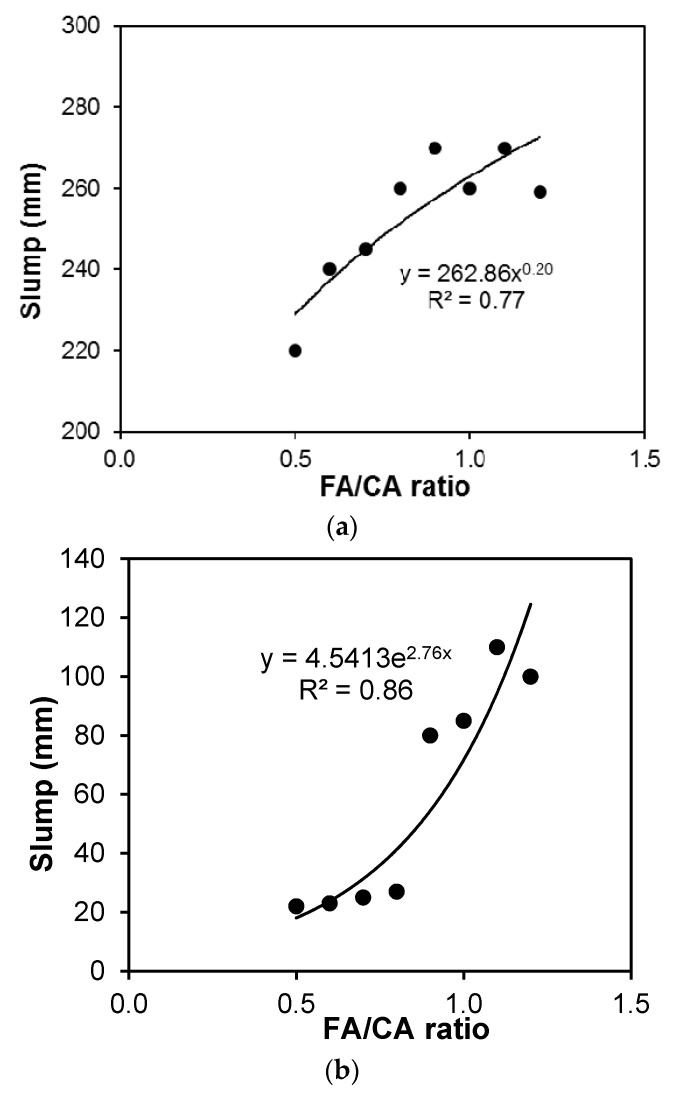
Slump vs. FA/CA ratio for concrete (**a**) slump of concrete without fiber; (**b**) slump of concrete with fiber.

**Figure 5:**
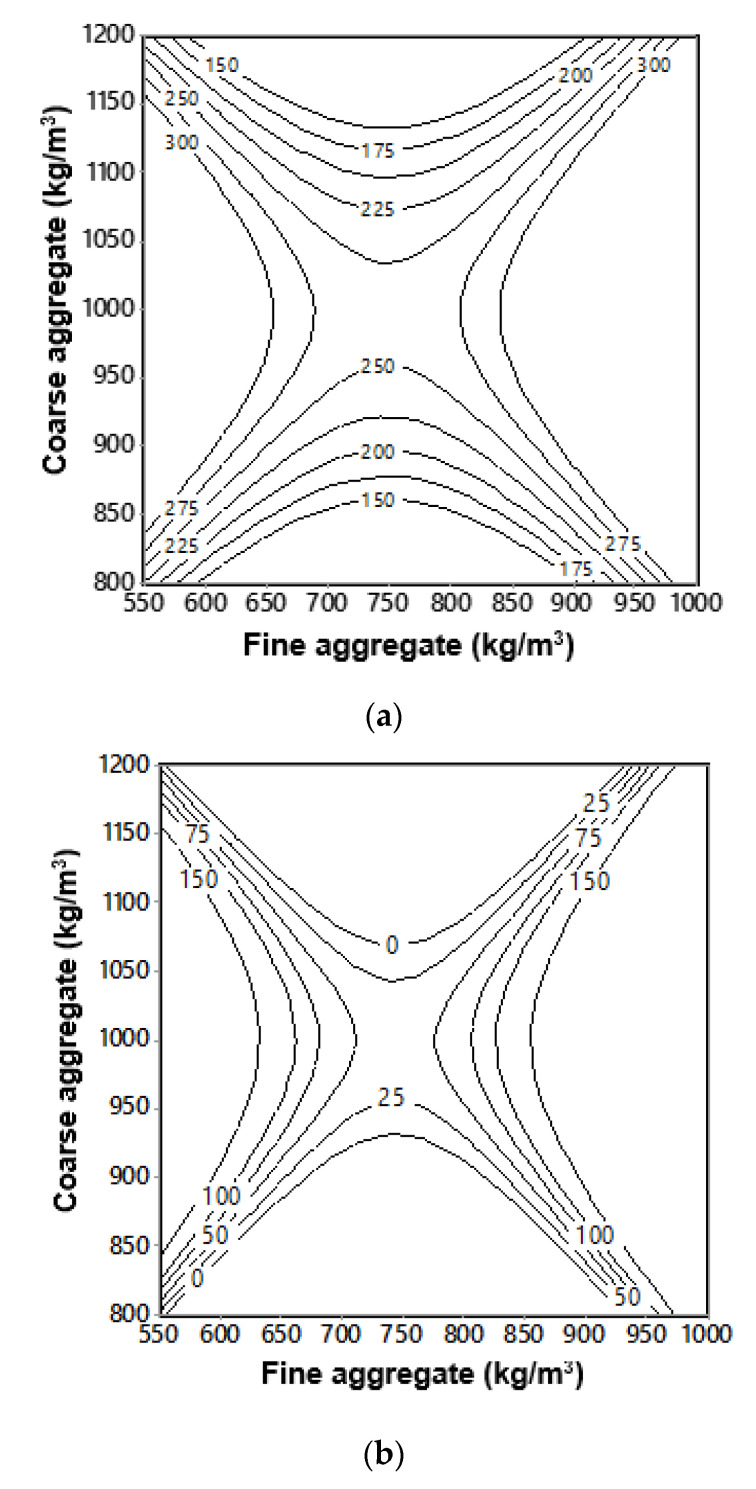
Isoresponse contours for slump (mm) (**a**) without fiber content; (**b**) with fiber content.

The measured slump was also plotted against the FA/CA ratio before and after adding steel fiber, as shown in Figure 4. The measured values of slump were used to establish models (using Equation (1)) for slump as follows:

(3) $$S_{wo-fiber}=260+46x_{1}+27x_{2}+182x_{1}^{2}-201x_{2}^{2};\;(R^{2}=0.96)$$

(4) $$S_{with-fiber}=46+95x_{1}+50x_{2}+321x_{1}^{2}-300x_{2}^{2};\;(R^{2}=0.90)$$

where $S_{wo-fiber}$ is slump without fiber (mm); $S_{with-fiber}$ is slump with fiber (mm); $x_{1}$ is the amount of fine aggregate (FA) content (%); $x_{2}$ is the amount of coarse aggregate (CA) content (%); and $R^{2}$ is the coefficient of determination, attributed to the model rather than to the random error. Equations (3) and (4) were plotted as interactive isoresponse contours for the prediction of slump as shown in Figure 5.

As seen from Figure 4 and Figure 5, the results revealed poor workability for mixtures with an FA/CA ratio of less than 0.8. It was also observed that the required dosage of SP drastically increased for FA/CA ratios above 0.8, to achieve a workable mixture. It is evident from the results that, as the percentage of the FA/CA ratio increases, the slump flow increases for the same range of flow of plain concrete. This may be attributed to the fact that an increase in fineness increases the specific area, and hence, a higher superplasticizer dosage is needed to wet the particles. This may also be attributed to finer particle to fiber contact, giving improved workability and flow. The relationship for the slump to FA/CA ratio is plotted and shows a good agreement of about 80% confidence with the results, as shown in Figure 4. This indicates that the slump for all FA/CA ratios was kept at higher values in high performance concrete (HPC), where the workability of HPC had a minimum value of 150 mm, but once the steel fibers were included in the concrete, the workability of the concrete was drastically affected by the addition of the fibers. It can also be observed in Figure 5 that the variation in FA and CA affected the slump of the SFRC. An exponential relationship fit the slump and FA/CA ratio best, as shown in Figure 4a,b. Higher FA/CA ratio mixtures achieved more slump, which was probably due to the availability of more fine aggregates, leading to a better fiber–matrix interaction and, thus, better workability.

Figure 5a,b were generated using Equations (3) and (4), respectively. These figures are based on the experimental results obtained for slump using various ratios of FA/CA, for concrete with and without fibers. With the information of the quantity of fine and coarse aggregate, the slump of concrete without fiber (Figure 5a) and for concrete with fiber (Figure 5b) can be estimate within the range of parameters investigated.

#### 3.1.2. Influence of Superplastizer on Concrete

**Figure 6:**
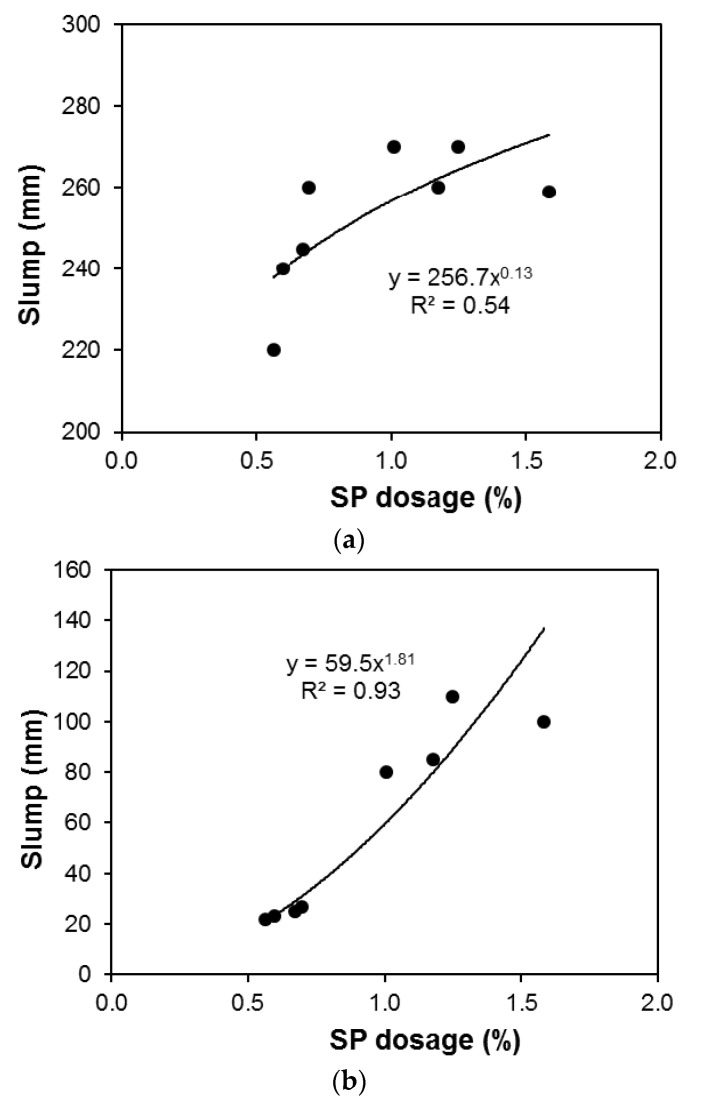
Influence of SP dosage on slump of concrete (**a**) without fibers; (**b**) with fibers.

Figure 6 shows the relationship between the slump and the SP dosage for the different mixtures prepared with different FA/CA ratios. These SP dosage values were plotted against the slump values of concrete, and it is evident from the graphs that the SP dosage increased the workability and led to a higher slump without fiber (Figure 6a), and with fiber (Figure 6b). This may be attributed to a higher surface area, due to more fine aggregates being available, causing a higher slump, as shown in Figure 6a,b. A power equation between the slump and the SP dosage was shown for the series of mixtures produced by different FA/CA ratios. As expected, the dosage of SP increased slump for both, concrete without fiber (Figure 6a), and with fiber (Figure 6b).

**Figure 7:**
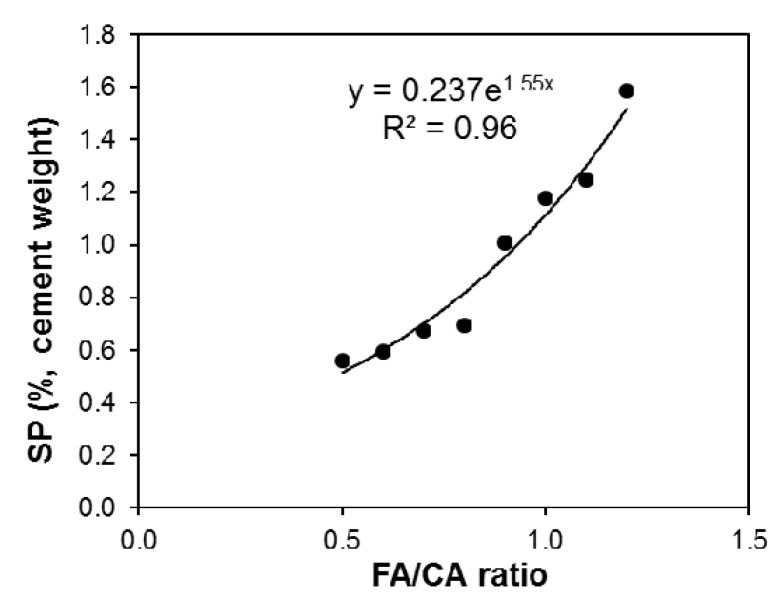
SP dosage (%) vs. FA/CA ratio for concrete.

Figure 7 shows that the governing relationship between the SP dosage and the FA/CA ratio is a power equation, because as the ratio of FA/CA increased, the dosage required for a certain level of concrete workability also increased. The additional SP produced with a higher concentration of fine aggregates is required for issues other than workability, for example, this increased dosage of SP is consumed for densification of the matrix by finer particles. This means that once a certain workability value is reached, additional SP will not improve the workability; rather, it will cause the reorganization of particles, forming a denser matrix system, whereas, for coarser particles, additional SP significantly increases the flowability of the matrix.

### 3.2. Compressive Strength

**Figure 8:**
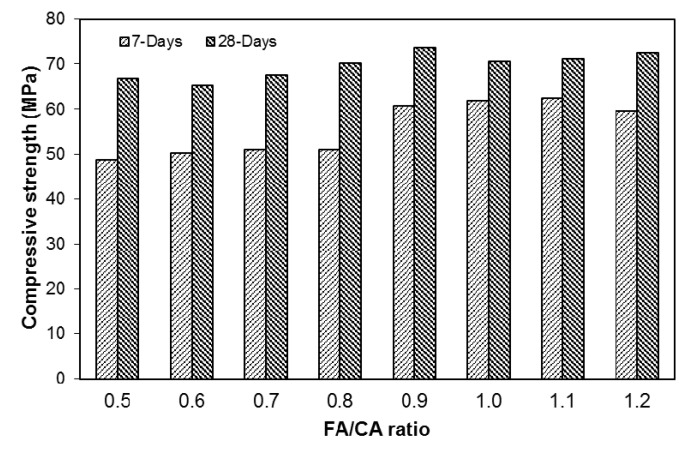
Compressive strength behavior of SFRC.

**Figure 9:**
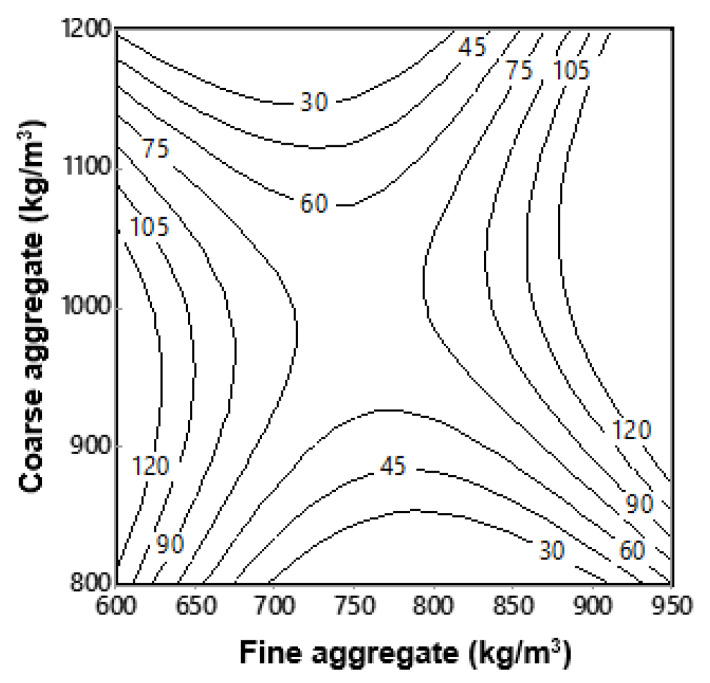
Isoresponse contours for compressive strength of SFRC.

A change in the FA/CA ratio affected the compressive strength of the concrete, as shown in Figure 8. As seen in the test results, there was no significant difference in the compressive strength at 7 and 28 days, especially when FA/CA < 0.9 (Figure 8). The 28-day compressive strength values were used to establish models (using Equation (1)) for compressive strength as follows:

(5) $$f_{c}^{\prime }=70-5x_{1}-1x_{2}+181x_{1}^{2}-67x_{2}^{2}+71x_{1}x_{2};\;(R^{2}=0.93)$$

where $f_{c}^{\prime }$ is the 28-day compressive strength (MPa); Equation (5) was plotted as interactive isoresponse contours for prediction of compressive strength, as shown in Figure 9.

The addition of fine aggregates influenced the compressive strength; however, the difference between the compressive strength of mixtures with low and higher FA/CA ratios was smaller (Figure 8). A higher fine aggregate content affected the compressive strength of mixtures with a constant w/c ratio. Although there was not a big difference in the compressive strength of concrete mixtures, there was a slight shift in the increment of strength after the FA/CA ratio increased from 0.8 to 0.9. The increase in the compressive strength with the rise in the FA/CA ratio was probably due to the filling of voids available in the concrete, due to the availability of more fine aggregate in the concrete; this also improved the workability of the SFRC. This statement proved to be evident as it is known that the availability of empty voids in the matrix causes a reduction in the mechanical properties of concrete; these voids can also decrease the workability of concrete mixtures [30].

Figure 9 was generated using Equation (5), and is based on the experimentally obtained values of compressive strength at 28 days for various ratios of FA/CA. This figure has prediction capability by having the information of the quantity of fine and coarse aggregates. The interactive responses of compressive strength shown in the figure are in good agreement with the experimentally obtained results.

### 3.3. Modulus of Elasticity

**Figure 10:**
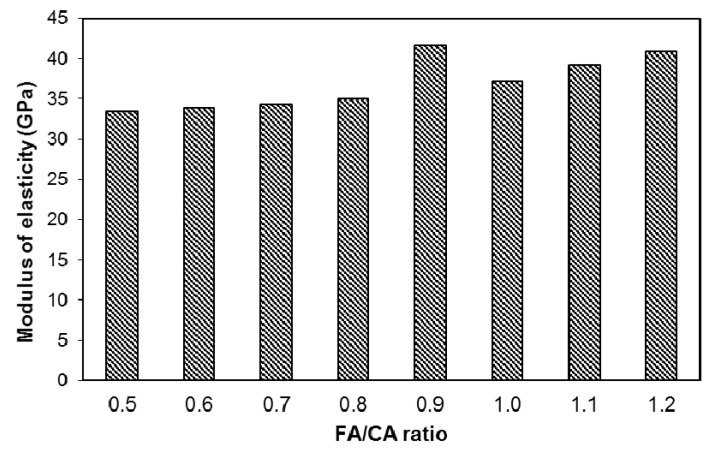
Modulus of Elasticity at 28 days of SFRC, with different FA/CA ratios.

The measured values of modulus of elasticity are presented in Figure 10. These values were used to establish models (using Equation (1)) for modulus of elasticity as follows:

(6) $$E_{c}=35.9+41.4x_{1}+38x_{2}+72x_{1}^{2}-70.8x_{2}^{2};\;(R^{2}=0.83)$$

where $E_{c}$ is modulus of elasticity (GPa).

**Figure 11:**
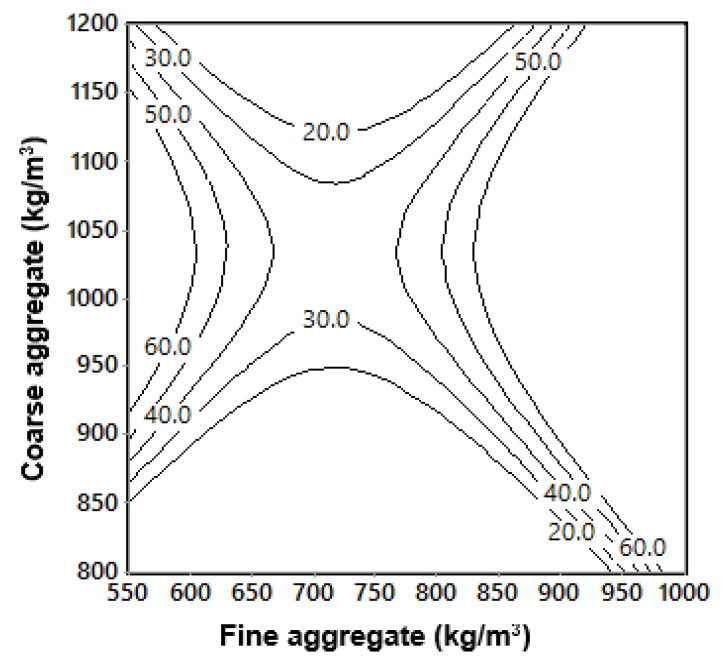
Isoresponse contours for modulus of elasticity of SFRC.

Equation (6) was plotted as interactive isoresponse contours for the prediction of modulus of elasticity, as shown in Figure 11. The modulus of elasticity showed a similar behavior as demonstrated by the compressive strength. At lower FA/CA ratios, the modulus of elasticity remained lower, but a higher value was achieved when the FA/CA ratio was 0.9. Isoresponse interactive contours of modulus of elasticity at 28 day of the SFRC were drawn using Equation (6), as shown in Figure 11. By knowing the quantity of FA and CA, the modulus of elasticity can be predicted from this figure. As expected, the patterns of the modulus of elasticity contours (Figure 11) are similar to those of the contours of compressive strength (Figure 9).

### 3.4. Load versus the Deflection

**Figure 12:**
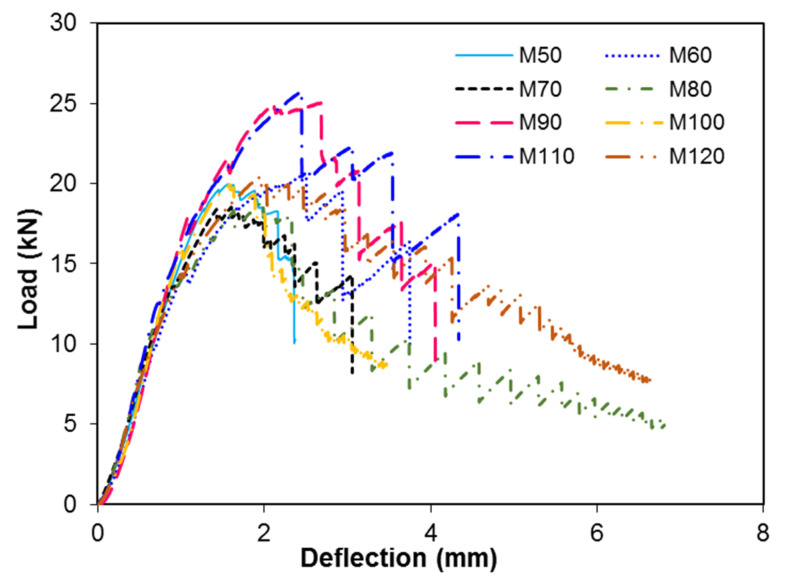
Load vs. deflection for different concrete mixtures.

**Figure 13:**
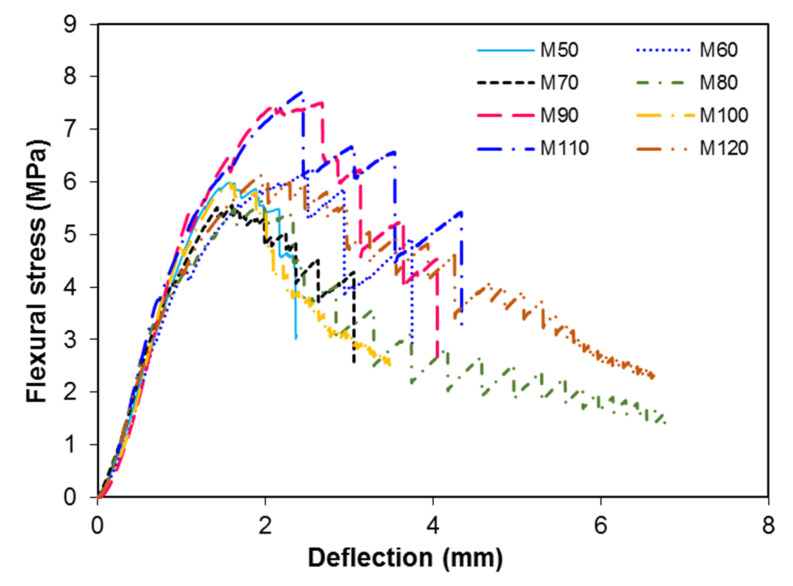
Flexural stress vs. deflection for different concrete mixtures.

The relationship between the load versus the deflection behavior of steel fiber reinforced concrete is shown in Figure 12. The flexural strength values were calculated using the following formula:

(7) $$\sigma =\left(\frac{3PL}{2bd^{2}}\right)$$

where σ is the flexural strength, *P* is the peak load, *L* is the span length of the prism, *b* is the width of the specimen, and *d* is the depth of the specimen. The variation in the flexural stress for different ratios of FA/CA is shown in Figure 13. The results demonstrated that the toughness of the matrix increased with an increase in the FA/CA ratio. It can be seen that the deflection behavior and flexural strength of the specimens were affected by the FA/CA ratio, for a constant water-to-cement ratio. As already mentioned in the previous section, the voids in the concrete are a major cause of the decrease in the mechanical properties of concrete. The flexural strength of concrete increases with the addition of fibers in concrete [11,13,14]. However, an increase in the flexural strength of SFRC was observed when the steel fiber content, and w/c ratio were kept constant but the FA/CA ratio was varied. The flexural strength was increased by up to 28% as the FA/CA ratio increased from 0.9 to 1.2 from a lower FA/CA ratio of 0.5.

The relationship of the load versus the deflection curve of the prisms was determined using the set up shown in the experiment, as shown in Figure 12. Steel fiber reinforced concrete can sustain a higher load than plain concrete. When a load was applied to the SFRC specimens, the failure first occurred the in concrete and after that the load was completely carried by the steel fibers, due to the debonding of fibers and complete pull-out of fibers. The steel fibers transferred the load to the concrete matrix by bridging the crack by means of bond stress. Therefore, SFRC does not experience sudden failure, like plain concrete; rather, it can continue to take load after the first crack or fracture of plain concrete.

### 3.5. Predictive Relationships

The predictive relationships for the calculation of the uniaxial compressive strength and the modulus of elasticity are mentioned in different codes such as Equation (8) [18], Equation (9) [31], and Equation (10) [32], as well as by other researchers, such as Equation (11) [33] and Equation (12) [34], and are as follows:

(8) $$E_{c}=4700\;\sqrt{f_{c}^{\prime }}$$

(9) $$E_{c}=6900\;\sqrt{f_{c}^{\prime }}+6900$$

(10) $$E_{c}=21,500\;\times \;\alpha_{\beta }\;\times \;{\left(\frac{f_{c}^{\prime }}{10}\right)}^{1/3}$$

(11) $$E_{c}=3.385\;\times \;10^{-5}\times \;w_{c}^{2.5}\;\times \;{\left(f_{c}^{\prime }\right)}^{0.325}$$

(12) $$E_{c}=16,191\;\sqrt{f_{c}^{,}}\;-13,540-2.41\;\times \;I_{sf}$$

where $E_{c}$ is the modulus of elasticity; $f_{c}^{\prime }$ is the compressive strength; $\alpha_{\beta }$ is a constant for the aggregate type, where 1.0 represents dense lime stone aggregate; $w_{c}$ is the density of concrete; and $I_{sf}$ is a reinforcing index (here, we used a value of 78.5).

**Figure 14:**
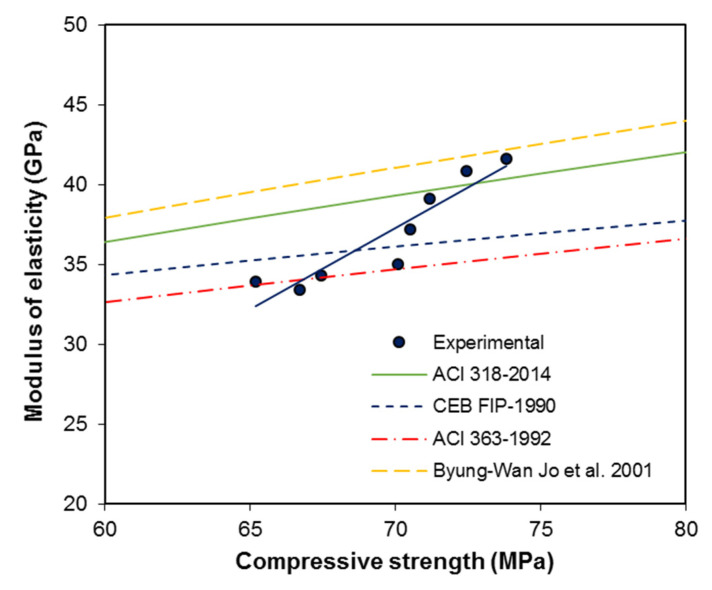
Compressive strength vs. modulus of elasticity of fiber reinforced concrete (FRC).

Figure 14 shows a comparison of the results with the different predictive formulas presented by different codes and researchers. Figure 14 shows that experimental results are in good agreement with the existing models; however, the results are more in agreement with ACI 363 [31], until a strength value of 70 MPa, whereas after 70 MPa, the experimental data points are more in agreement with ACI 318 [18], CEB-FIP [32], and Byung-Wan Jo et al. [34]. A shift in the data series can be clearly observed, so there may be a series data shift at a compressive strength of around 70 MPa. This should be further explored in another study. From these tests, the increased elastic modulus value may be attributed to the addition of fine material in the preparation of concrete mixtures. This fine material may have caused a reduction in micro cracks, and provided adhesive force for the cement paste and aggregate.

The relationships between the uniaxial compressive strength and flexural strength available in the present literature such as Equation (13) [18], Equation (14) [33], Equation (15) [35], Equation (16) [31], and Equation (17) [31], and are as follows:

(13) $$f_{t}=0.62f_{c}^{0.5}$$

(14) $$f_{t}=0.44f_{c}^{0.5}$$

(15) $$f_{t}=0.39f_{c}^{0.59}$$

(16) $$f_{t}=0.259f_{c}^{0.843}$$

(17) $$f_{t}=0.94f_{c}^{0.5}$$

**Figure 15:**
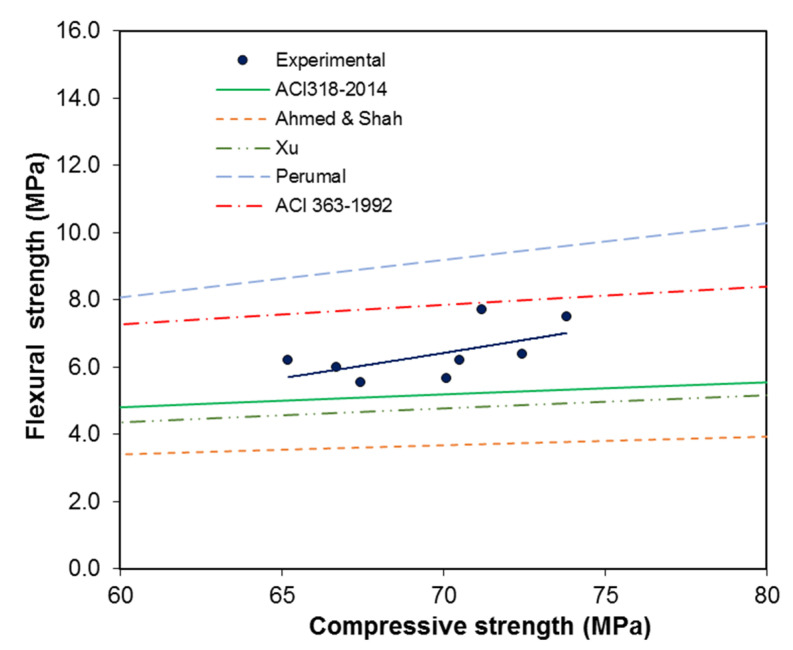
Compressive strength vs. flexural strength of FRC.

The generalized forms of these relationships can be seen in Equation (18). A comparison between the experimental data and the predictive curves is shown in Figure 15.

(18) $$f_{t}=nf_{c}^{x}$$

With an increase in compressive strength, the experimental data fit between the ACI 318 [18] and ACI 363 [31] relationships, as shown in Figure 14. The results show that the experimental results are at values above or near those of the ACI 318 [18] predictive equation. This means that the ACI 318 [18] predictive equation underestimates the flexural strength of SFRC.

The results revealed that the curves proposed by Xu and Shi [35] and Perumal [36] underestimate the flexural strength compared to the experimental data points. The values of the experimental data were well above those of Ahmed and Shah’s [33] predicted equation. This deviation may be due the fact that the experimental data points were for high-strength concrete with hook ended fibers. Figure 15 shows that the flexural strength of FRC concrete increases comparably with an increase in the compressive strength. The results revealed that the flexural strength deviates more from empirical relations with an increase in compressive strength, which could be due to the use of fiber reinforced high-strength concrete.

**Figure 16:**
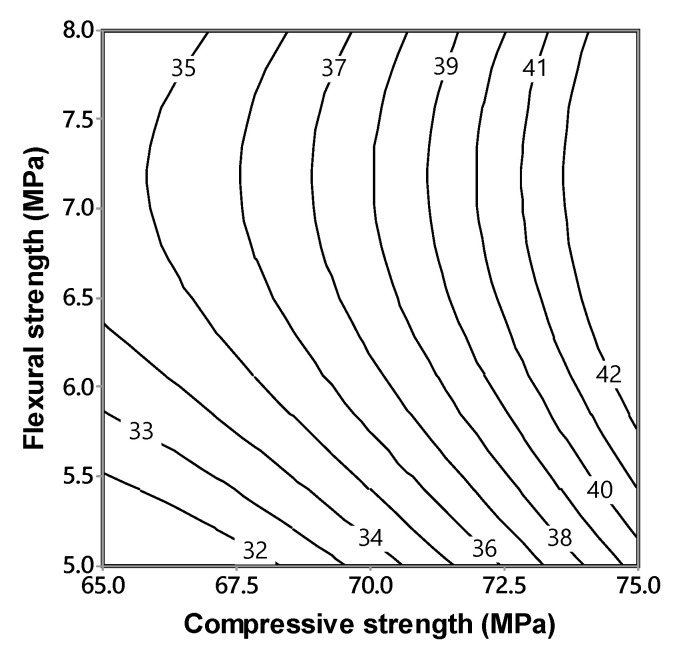
Isoresponse contours for modulus of elasticity vs. compressive strength and flexural strength at 28 days.

The modulus of elasticity values were used to establish models (using Equation (1)) using the measured values of compressive strength and flexural strength, as follows:

(19) $$E_{c}=37.2+3.76f_{c}^{\prime }+1.13\sigma +0.99f_{c}^{\prime }{}^{2}-1.11\sigma^{2};\;(R^{2}=0.95)$$

where $E_{c}$ is modulus of elasticity at 28 days (GPa); $f_{c}^{\prime }$ is compressive strength at 28 days (MPa) and σ is flexural strength at 28 days (MPa). Equation (19) was plotted as predictive isoresponse contours for modulus of elasticity, as shown in Figure 16.

Figure 16 was generated using the Equation (19) and is based on the experimentally obtained results of compressive strength and flexural strength at 28 days. Using these values, interactive contours of modulus of elasticity were plotted. From this figure these contours show the trends influenced by both compressive strength and flexural strength. The correlation of the relationship is significantly high, and has the potential capability to predict the values within the scope of the experimental domain.

## 4. Conclusions

The main conclusions of this investigation can be summarized as follows:

- The addition of fiber contents greatly influences the workability of SFRC, and caused a reduction in slump. The material movement was reduced due to the addition of fibers, which act as a barrier to the movement of coarse aggregate.
- An increase in the FA/CA ratio affects the workability of SFRC, however, it does not have negative effect on its compressive strength.
- FA/CA ratio above 0.8 showed better flowability in the fresh state of SFRC, however, FA/CA ratio above 0.9 needed excessive superplasticizer to maintain flowability.
- Higher values of FA/CA ratio increased both compressive strength and flexural strength of SFRC, up to 10% and 28%, respectively.
- Steel fibers transferred the load to the concrete matrix by bridging the crack by means of bond stress, and continued to take load after the first crack or fracture of plain concrete.
- Experimental values of compressive strength and flexural strength showed good agreement with the available existing prediction equations, however, modulus of elasticity demonstrated slightly higher values.
- Experimentally obtained measurements enabled the establishment of isoresponse interactive equations and contours, which have prediction capabilities within the scope of the investigation domain.
- FA and CA contribute up to 75% of total concrete volume, therefore, balancing the usage of FA and CA plays a vital role in determining the performance and quality of the concrete.

## Figures and Tables

**Table 1:** The chemical composition and physical properties of OPC (Ordinary Portland cement).

Elemental Oxide (%)							Loss on Ignition (%)	Specific Gravity	Fineness (m^2^/kg)
SiO_2_	Al_2_O_3_	Fe_2_O_3_	CaO	MgO	SO_3_	Na_2_O			
20.2	5.49	4.12	65.43	0.71	2.61	0.26	1.38	3.14	373

**Table 2:** Characteristic physico-mechanical properties of steel fibers.

Aspect Ratio	Length (mm)	Diameter (µm)	Tensile Strength (MPa)	Young’s Modulus (GPa)
80	60	750	1250	210

**Table 3:** Mixes proportions for SFRC (steel fiber reinforced concrete) mixes.

Specimen ID	FA/CA Ratio	Fine Aggregate (kg/m^3^)	Coarse Aggregate (kg/m^3^)	Water (kg/m^3^)	SP ^1^ (mL)
M50	0.5	580	1170	171.19	117
M60	0.6	660	1100	170.70	124
M70	0.7	730	1040	170.44	140
M80	0.8	560	980	170.0	120
M90	0.9	835	930	169.70	210
M100	1.0	880	880	169.37	245
M110	1.1	925	840	169.14	260
M120	1.2	965	800	168.88	330

^1^ Structure superplasticizer.

**Table 4:** The properties of fresh concrete.

Specimen ID	Fresh Properties			
	Slump ^1^ (mm)	Slump ^2^ (mm)	Temperature (°C)	Unit Weight (kg/m^3^)
M50	220	22	23.9	2306
M60	240	23	23	2403
M70	245	25	23.2	2343
M80	260	27	23.3	2356
M90	280	80	21.7	2276
M100	260	85	22.3	2260
M110	270	110	23.7	2266
M120	240	100	21.8	2353

^1^ Measurement of slump without Steel Fibers; ^2^ Measurement of slump with Steel Fibers.

## References

[1] Sung-Hoon K., Sung-Gul H., Moon J. (2020). Performance comparison between densified and undensified silica fume in ultra-high performance fiber-reinforced concrete. Materials.

[2] Grünewald S., Walraven J.C. (2001). Parameter—Study on the influence of steel fibers and coarse aggregate content on the fresh properties of self—Compacting concrete. Cem. Concr. Res..

[3] Sahmaran M., Christianto H.A., Yaman I.O. (2006). The effect of chemical admixtures and mineral additives on the properties of self-compacting mortars. Cem. Concr. Res..

[4] Benabed B., Kadri E., Azzouz L., Kenai S. (2012). Properties of self-compacting mortar made with various types of sand. Cem. Concr. Res..

[5] Boulekbache B., Hamrat M., Chemrouk M., Amziane S. (2010). Flowability of fiber-reinforced concrete and its effect on the mechanical properties of the material. Constr. Build. Mater..

[6] Khayat K.H. (1999). Workability, testing and performance of self-compacting concrete. ACI Mater. J..

[7] El-Dieb A.S., Taha M.M.R. (2012). Flow characteristics and acceptance criteria of fiber-reinforced self-compacted concrete (FR-SCC). Constr. Build. Mater..

[8] Khayat K.H., Roussel Y. (2000). Testing and performance of fiber-reinforced, self-consolidating concrete. Mater. Struct. Matér. Constr..

[9] Mostafa S.A., Faried A.S., Farghali A.A., EL-Deeb M.M., Tawfik T.A., Majer S., Elrahman M.A. (2020). Influence of nanoparticles from waste materials on mechanical properties, durability and microstructure of UHPC. Materials.

[10] Siwiński J., Szcześniak A., Stolarski A. (2020). Modified formula for designing ultra-high-performance concrete with experimental verification. Materials.

[11] Ulas M.A., Alyamaç K.E., Ulucan Z.C. Assessment of the Behavior of Steel-Fiber Reinforced Concrete Produced with Different Ratios of Fine-to-Coarse Aggregate. Proceedings of the 4th International Conference on Sustainable Construction Materials and Technologies.

[12] ACI Committee 544.1R (2005). State-of-the-Art Report on Fiber Reinforced Concrete.

[13] Banthia N., Sappakittipakorn M. (2007). Toughness enhancement in steel fiber reinforced concrete through fiber hybridization. Cem. Concr. Res..

[14] Tadepalli P.R., Mo Y.L., Hsu T.T.C. (2013). Mechanical properties of steel fibre concrete. Mag. Concr. Res..

[15] Banthia N. (1990). A study of some factors affecting the fiber–matrix bond in steel fiber reinforced concrete. Can. J. Civ. Eng..

[16] Chenkui H., Guofan Z. (1995). Properties of Steel Fibre Reinforced ConcretebContaining Larger Coarse Aggregate. Cem. Concr. Compos..

[17] Kim J.J., Kim D.J., Kang S.T., Lee J.H. (2012). Influence of sand to coarse aggregate ratio on the interfacial bond strength of steel fibers in concrete for nuclear power plant. Nucl. Eng. Des..

[18] ACI 318-19 (2019). Building Code Requirements for Structural Concrete and Commentary.

[19] Fantilli A.P., Cavallo A.D., Pistone G. (2015). Fiber-reinforced lightweight concrete slabs for the maintenance of the Soleri Viaduct. Eng. Struct..

[20] Lee S.-J., Yoo D.-Y., Moon D.-Y.K., Sung-Gul H., Moon J. (2019). Effects of Hooked-End Steel Fiber Geometry and Volume Fraction on the Flexural Behavior of Concrete Pedestrian Decks. Appl. Sci..

[21] Neves R.D., de Almeida J.C.O.F. (2005). Compressive behaviour of steel fibre reinforced concrete. Struct. Concr..

[22] Abbass W., Khan M.I., Murad S. (2018). Evaluation of mechanical properties of steel fiber reinforced concrete with different strengths of concrete. Constr. Build. Mater..

[23] ACI 544.3R-08 (2008). Guide for Specifying, Proportioning, and Production of Fiber-Reinforced Concrete.

[24] ASTM C150M-20 (2020). Standard Specification for Portland Cement. American Society for Testing and Materials.

[25] ASTM C494M-19 (2019). Standard Specification for Chemical Admixtures for Concrete. American Society for Testing and Materials.

[26] ASTM C33M-18 (2018). Standard Specification for Concrete Aggregates. American Society for Testing and Materials.

[27] ASTM C31M-19a (2019). Standard Practice for Making and Curing Concrete Test Specimens in the Field. American Society for Testing and Materials.

[28] ASTM C469M-18 (2018). Standard Test Method for Static Modulus of Elasticity and Poisson’s Ratio of Concrete in Compression. American Society for Testing and Materials.

[29] Ryan B.F., Joiner B.L., Cryer J.D. (2012). Minitab Handbook, Release 16.

[30] Neville A.M. (1995). Properties of Concrete.

[31] ACI Committee 363R-97 (1997). State-of-the-Art Report on High Strength Concrete.

[32] CEB-FIP Model Code for Concrete Structures (1991). Evaluation of the Time Dependent Behavior of Concrete.

[33] Ahmad S.H., Shah S.P. (1985). Structural properties of high strength concrete and its applications for precast prestressed concrete. PCI J..

[34] Jo B.-W., Shon Y.-H., Kim Y.-J. (2001). The Evalution of Elastic Modulus for Steel Fiber Reinforced Concrete. Russ. J. Nondestruct. Test..

[35] Xu B.W., Shi H.S. (2009). Correlations among mechanical properties of steel fiber reinforced concrete. Constr. Build. Mater..

[36] Perumal R. (2015). Correlation of Compressive Strength and Other Engineering Properties of High-Performance Steel Fiber–Reinforced Concrete. J. Mater. Civ. Eng..

